# Accuracy of AFM cantilever thermal calibration in fluid of varying viscosities

**DOI:** 10.1088/1361-6501/aebb53

**Published:** 2026-11-10

**Authors:** Abideen O Ayangbemi, David R Jacobson

**Affiliations:** 1 Department of Chemistry, Clemson University, Clemson, SC 29634, United States of America; 2 Medical Biophysics Graduate Program, Clemson University, Clemson, SC 29634, United States of America

**Keywords:** atomic force microscopy, single molecule force spectroscopy, AFM cantilever calibration

## Abstract

Thermal calibration of atomic force microscopy (AFM) cantilever spring constant, *k*, is routinely performed by fitting the thermal displacement power spectral density (PSD) to a simple harmonic oscillator (SHO) model. Experiments performed in liquid present the choice of whether to also calibrate in liquid or to pre-calibrate in air, where reduced hydrodynamic drag produces a sharper resonance peak. It remains an open question whether this yields a more accurate calibration. Here we use a hydrodynamic model introduced by Sader and later extended by Bellon to generate thermal PSDs for two cantilevers of differing known stiffness across four media of increasing viscosity. We then fit each generated PSD with the SHO model used in experimental calibration (under various parameters and conditions). Iteratively fitting in the vicinity of the resonance peak, with logarithmic weighting and a fitted noise term, recovers the known spring constant to within 3% in every medium including the most viscous, where the fundamental mode of the softer cantilever is overdamped. We use this analysis to identify an optimal fitting strategy—in terms of window size, window center, and weighting—for experimentally reasonable cantilever properties and describe a general procedure for determining these parameters for other cantilevers. We show that the optimal fit-window width is not universal but can be determined for any cantilever and medium by applying the same analysis with different parameters. To confirm these results, we experimentally calibrated the same two types of cantilevers in air and liquid and obtained *k* values that agreed closely, despite several-fold changes in resonance frequency and quality factor upon immersion. These results demonstrate that AFM cantilevers intended for liquid-phase measurements can be reliably calibrated *in situ*, without recourse to a separate calibration in air.

## 1. Introduction

Atomic force microscopy (AFM) is a powerful tool for applying and measuring mechanical forces at the single-molecule level, enabling direct probing of biomolecular mechanics. AFM-based single-molecule force spectroscopy (SMFS) has been applied across a wide range of biological systems, including protein folding, [1–3] ligand-receptor binding, [4, 5] DNA and polymer elasticity, [6, 7] lipid bilayer mechanics, [8] and cell adhesion and mechanotransduction [9]. To retain biological relevance, AFM measurements of these systems must be performed in aqueous buffer.

In an SMFS experiment, the tip of a cantilever is brought into contact with a sample (e.g. a single protein molecule tethered to a surface or embedded in a lipid bilayer) and then retracted while the cantilever deflection is measured (figure 1(a)). The cantilever behaves as a linear spring obeying Hooke’s law, *F* = *kx*, where *k* is the cantilever spring constant and *x* is the cantilever deflection. The deflection is measured using the optical lever method, where the displacement of a laser beam reflected from the cantilever onto a photodetector is recorded as a voltage signal and converted to deflection through calibration of the inverse optical lever sensitivity (invOLS) (figure 1(b)) [10, 11]. InvOLS is most directly calibrated by pressing the cantilever tip against a rigid surface, where the photodetector voltage tracks the known piezo displacement and the invOLS is obtained as the inverse of the slope of the deflection signal versus piezo displacement. This invOLS depends on the index of refraction of the medium, the cantilever geometry, the optical setup, the laser power, and other factors. Therefore, invOLS must be redetermined whenever the cantilever is reloaded into the instrument or whenever any aspect of the optical alignment is changed; invOLS values for the same cantilever in air and liquid can differ substantially. Equally important to the invOLS calibration is the calibration of *k*, which is the focus of this work (figure 1(c)). Uncertainties in the spring constant directly propagate into all derived quantities, including measured forces, contour lengths, energy landscapes, and kinetic parameters extracted from single-molecule experiments [12]. Accurate calibration of *k* has therefore long been recognized as a central requirement for quantitative AFM force measurements, motivating the development of reference calibration methods, traceable force standards, and formal uncertainty analyses [13, 14].

**Figure 1.**
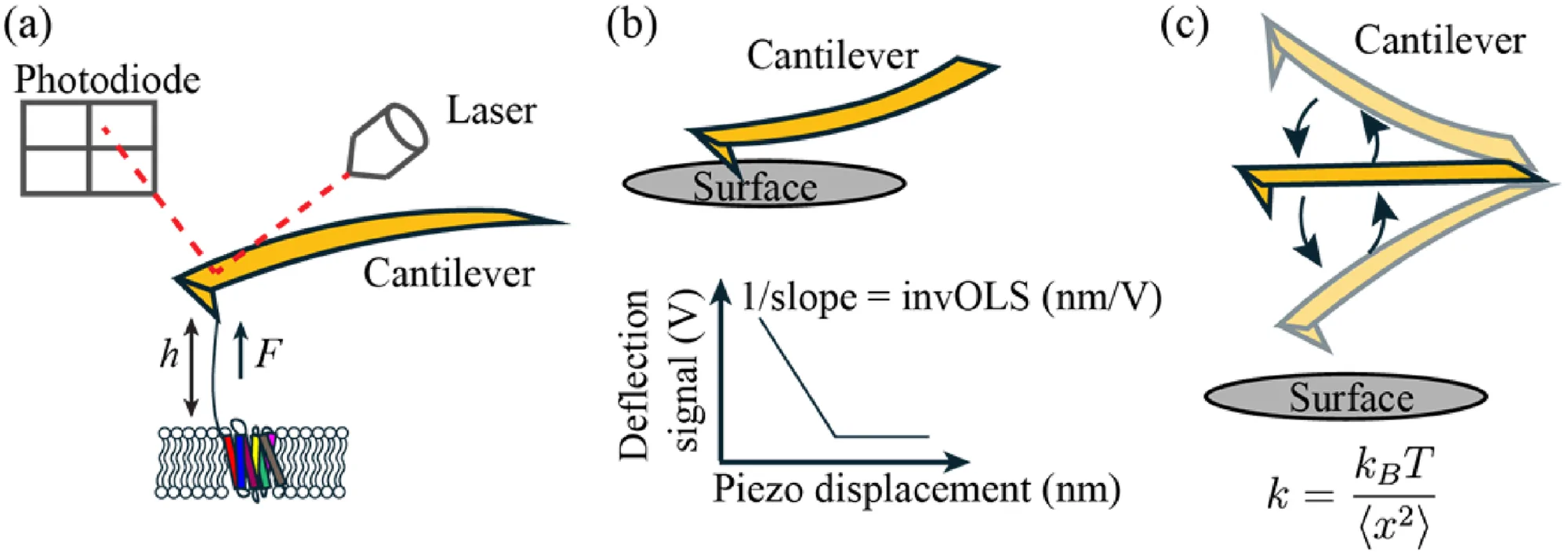
(a) Schematic of an AFM-based single-molecule force spectroscopy (SMFS) assay of a membrane protein. A cantilever tip is brought into contact with a membrane protein in a lipid bilayer and retracted while deflection is monitored via the optical lever method. A laser beam reflected from the back of the cantilever onto a position-sensitive photodiode records the deflection signal, from which the applied force is recovered via calibrated inverse optical lever sensitivity (invOLS) and spring constant (*k*). (b) Schematic of invOLS calibration. The cantilever tip is pressed against a hard surface and the deflection signal is recorded as a function of piezo displacement. The inverse of the slope of this curve gives the invOLS. (c) Schematic of *k* calibration via the thermal noise method. With the cantilever freely suspended above the surface, thermal fluctuations drive random motion. The equipartition theorem relates the variance of this motion to *k*. In practice, the amplitude is recorded and analyzed in the frequency domain to extract *k*.

The mechanical response of an AFM cantilever is conventionally modeled as a damped harmonic oscillator characterized by three parameters: the spring constant *k*, the resonance frequency *f*_0_, and the quality factor *Q* [15, 16]. The thermal motion of the cantilever can be characterized in the frequency domain through its power spectral density (PSD), which describes how the cantilever’s thermal displacement energy is distributed across frequencies (figure 2). For an underdamped oscillator (*Q* > ½), the PSD is sharply peaked at *f*_0_, with a flat white-noise plateau at *f* < *f*_0_ and *f*^−4^ decay at *f* > *f*_0_ (figure 2(a)). As damping increases and *Q* decreases toward and below the critical damping condition (*Q* = ½), this peak progressively broadens and weakens, disappearing entirely once the oscillator is overdamped, although the total area under the PSD remains constant to satisfy the equipartition theorem. The PSDs in figure 2 assume that the damping experienced by the cantilever is frequency-independent. In actual experiments, a cantilever immersed in a viscous fluid experiences hydrodynamic loading that varies with oscillation frequency, which modifies the shape of the thermal PSD in ways that the damped harmonic oscillator model cannot fully capture.

**Figure 2.**
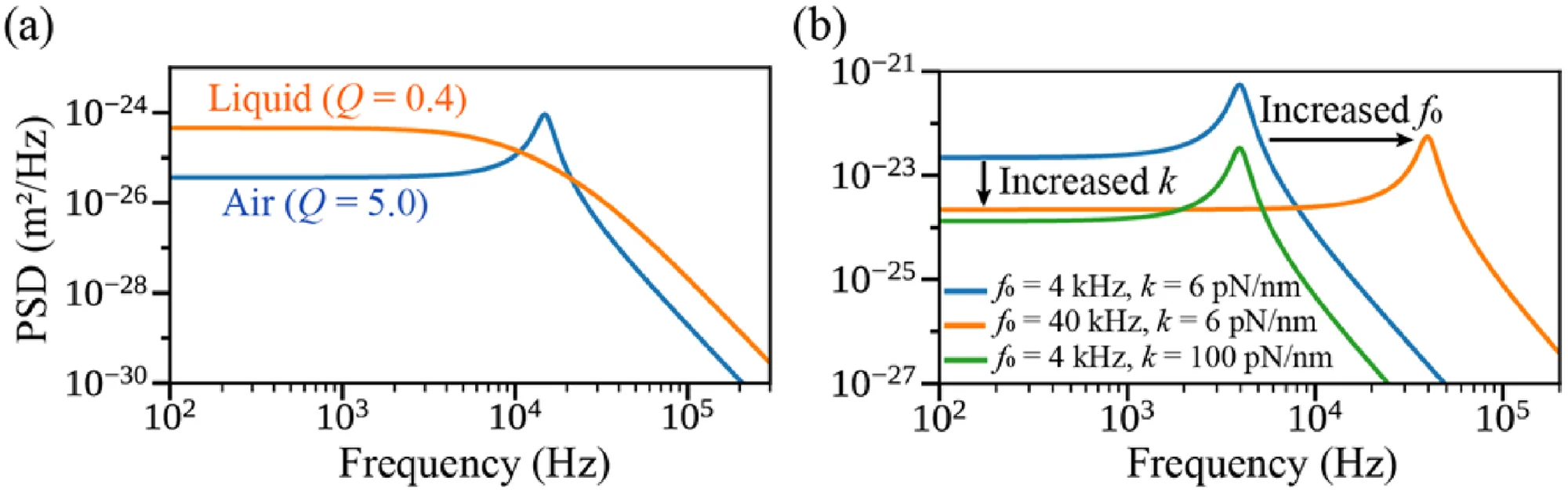
Analytical power spectral density (PSD) of a single-mode damped harmonic oscillator (equation (2)), illustrating how quality factor (*Q*), spring constant (*k*), and resonance frequency (*f*_0_) each shape the thermal noise spectrum under the idealized assumption of frequency-independent damping. (a) PSD for an underdamped oscillator in air (*Q* = 5.0, blue) and an overdamped oscillator in liquid (*Q* = 0.4, orange), with *f*_0_ and *k* held fixed. The air spectrum exhibits a sharp, well-resolved resonance peak, while the liquid spectrum is broad and shallow, with most of its spectral weight shifted to frequencies below *f*_0_. (b) PSD curves illustrating the independent effects of increasing *k* (blue to green, same *f*_0_) and increasing *f*_0_ (blue to orange, same *k*) at fixed *Q*. Increasing *k* lowers the low-frequency plateau without shifting the peak position, whereas increasing *f*_0_ shifts the entire resonance to higher frequency while lowering the plateau height.

Among the available cantilever calibration methods, the thermal noise-based approach is widely used due to its firm theoretical foundation, non-destructive nature, applicability across a wide range of cantilever stiffnesses and environmental conditions, and the fact that the fitting procedure itself requires no prior knowledge of cantilever geometry [16–19]. It is particularly relevant to high-time-resolution measurements of biomolecules using ultrashort cantilevers or cantilevers with modified geometries, since the alternative method of Sader *et al* [20–22] requires precise knowledge of the cantilever’s plan dimensions that become difficult to measure in these cases. The thermal method is grounded in the equipartition theorem, which assigns to each quadratic degree of freedom of a thermal system an average energy of $\frac 1{2}{k_\mathrm{B}}T$, where *k*_B_ is Boltzmann’s constant and *T* is the absolute temperature. The spring constant can be written in terms of the variance of cantilever deflection 〈*x^2^*〉:

\begin{equation*}k = \frac{{k_\mathrm{B}}T}{ \langle {x^2} \rangle}.\end{equation*}

Because the overall variance of time-series data can also be biased by undersampling or parasitic noise, the thermally calibrated value of *k* is, in practice, obtained by fitting the PSD (*S_xx_*) of the freely fluctuating cantilever to the expected Lorentzian functional form of a damped harmonic oscillator: [15, 16, 23]

\begin{equation*}{S_{xx}}\left(f\right) = \frac{2{k_\mathrm{B}}T}{\pi k{f_0}Q}\cdot \frac 1{{\left[ {\left( \frac f{{f_0}}\right) ^2}-1\right] ^2}+{\left( \frac f{{f_0}Q}\right) ^2}}+{S_\mathrm{w}}.\end{equation*}

Here *S*_w_ is an additional term that can be added to model frequency-independent white-noise background deriving from detection noise or other contributions uncorrelated with the cantilever’s thermal motion.

Because it involves three fit parameters—or four including *S*_w_—there is a concern that fitting equation (2) will not yield an accurate thermal calibration if the experimental PSD does not contain enough information to constrain all parameters. This is a special concern in liquid, where damping suppresses the resonance peak (figure 2(a)). Conversely, invOLS calibration in liquid can be more facile, especially for soft cantilevers, because aqueous solution screens electrostatic interactions between the cantilever and surface. This allows the tip to be brought in contact with the surface without the snap-to-contact and adhesion artifacts that can make it difficult to accurately measure the surface-contact slope in air (figure S1). There are also practical advantages to calibrating a cantilever immediately before it is used or even from thermal fluctuations recorded during the single-molecule assay itself.

These considerations raise a fundamental question: should cantilevers intended for liquid experiments be calibrated in air beforehand or calibrated *in situ* within the aqueous measurement environment? This choice is unique to AFM. While thermal PSD fitting is also used to calibrate optical traps and magnetic tweezers, [24] those traps cannot be moved between media in the way a cantilever can. It is possible to address this question using theory to predict the PSDs of cantilevers with known parameters, which can then be fit with equation (2). Such an approach, however, requires an accurate physical model of cantilever thermal oscillations in liquid.

A physically complete description of the cantilever’s thermal motion must account for its frequency-dependent drag arising from its complex hydrodynamic function. Such a description was developed by Sader [22] and later elaborated by Bellon [25]. For a cantilever idealized as a thin, long rectangular beam immersed in a viscous fluid, Sader showed that the hydrodynamic loading exerted by the surrounding fluid is captured by a dimensionless hydrodynamic function *Γ*(*ω*), whose real part accounts for the added mass of fluid entrained by the moving beam and whose imaginary part accounts for the viscous drag it experiences [22]. Because *Γ*(*ω*) varies with frequency, the effective mass and damping of the cantilever are frequency-dependent, in contrast to the assumption underlying equation (2). Building on this framework, Bellon applied the fluctuation-dissipation theorem to each flexural mode of the cantilever to derive the thermal displacement PSD of a real beam of specified geometry in a fluid of specified density and viscosity [25]. The resulting PSD departs qualitatively from the Lorentzian form of equation (2) in lacking a flat low-frequency plateau and exhibiting a more-gradual high-frequency rolloff.

Here we compare the accuracy of thermal calibration of *k* in air and liquid conditions—and in different viscosities—by using the Sader-Bellon model to predict PSDs of cantilevers with known parameters; we then fit these PSDs with the Lorentzian equation used in experimental fitting (equation (2)). We are not aware of prior studies employing this approach, which have mostly compared different experimental calibration methods [19, 23, 26, 27] or calibration under different experimental conditions [28, 29]. Less systematic attention has been given to the bias introduced by the mathematical model used to fit the thermal spectrum, particularly under strongly damped liquid conditions. An advantage of our approach is that the underlying value of *k* in the generated PSDs is known *a priori* and thus can be used as a basis for calculating bias in the fit results, whereas the stiffness of real cantilevers can vary significantly from the manufacturer’s nominal value. We were thus able to optimize the fitting in terms of width, weighting, and number of iterations to minimize the bias. We tested these optimized conditions, as well as our conclusion that cantilevers *can* be accurately calibrated in liquid, through test experiments on two types of cantilevers relevant to SMFS. Although we focus on SMFS, the results are relevant to any AFM measurement that requires calibrated force in liquid, such as nanoindentation of cells and soft materials, colloidal-probe force measurements, and mechanobiology [30–32].

## 2. Methods

### 2.1. Sader-Bellon viscous PSD model

Thermal displacement PSDs were generated using the Sader-Bellon model, which describes a cantilever as a continuous elastic beam, clamped at one end and free at the other, immersed in a viscous fluid. The Sader-Bellon model assumes a uniform rectangular beam that is long relative to its width, so the simulated PSDs represent cantilevers that satisfy these geometric assumptions. Commercial probes may depart from this idealization in their shape, dimensional uniformity, or the additional mass contributed by the tip. These assumptions pertain to the generation of the model PSDs and are distinct from the subsequent approximation of fitting those PSDs with the simple harmonic oscillator (SHO) model, whose accuracy is evaluated separately in this work. The PSD contribution of flexural mode *n* is given by: [25]

\begin{equation*}{S_n}\left(\omega \right) = \frac{2{k_\mathrm{B}}T}{\pi \omega}\mathfrak{I}\left( \frac 1{\frac k{3}{C_n^4}-{{\left[{m_\mathrm{c}}+{m_\mathrm{f}}\Gamma \left(\omega \right)\right]\omega}^2}}\right) ,\end{equation*}

where *ω* is the angular frequency, $\mathfrak{I}$ (·) is the imaginary part of the argument, *m*_c_ is the cantilever mass, and ${m_\mathrm{f}} = \pi \rho L{b^2}/4$ is the mass of fluid entrained by the cantilever’s motion, with *ρ* the fluid density, *L* the cantilever length, and *b* the cantilever width. The modal stiffness ${k_n} = \left( \frac k{3}\right) {C_n^4}$, where *C_n_* is the *n*th positive root of the clamped-free eigenvalue equation $1+\cos ({C_n})\cosh ({C_n}) = 0$, solved numerically. The first two roots, *C*_1_ = 1.8751 and *C*_2_ = 4.6941, were used. Because the flexural modes are orthogonal and their thermal fluctuations uncorrelated, the deflection PSD at any point along the beam is the sum of the individual modal PSDs weighted by the squared mode shape at that point [25]. Evaluating at the free end (*x* = 1 in equation 24 of Sader [22]) and noting that the squared modal amplitude ${|{\varphi_n}(1)|^2}$ equals 4 for every mode of a clamped-free beam, the first two modes contribute with equal weight. A factor of 2π converts the angular frequency PSD of equation (3) to the ordinary-frequency PSD as:

\begin{equation*}{S_\omega}\left(f\right) = 2\pi \left[{|{\varphi_1}\left(1\right)|^2}{S_1}\left(2\pi f\right)+{|{\varphi_2}\left(1\right)|^2}{S_2}\left(2\pi f\right)\right].\end{equation*}

The hydrodynamic function ${\Gamma} (\omega ) = {\Omega}(\omega )\cdot {{\Gamma}_{\mathrm{circ}}}(\omega )$ captures the frequency-dependent loading of the surrounding fluid on the beam in terms of the exact hydrodynamic function for an infinitely long circular cylinder, Γ_circ_(*ω*), and the rectangular correction factor *Ω*(*ω*), parameterized in terms of the oscillatory Reynolds number $Re = \rho \omega {b^2}/4\eta$ (equations 18 and 21 of Reference 22; *η* the fluid viscosity).

PSDs were generated for four fluid media spanning more than three orders of magnitude in dynamic viscosity: air (*ρ* = 1.18 kg m^−3^, *η* = 1.84 ×10^−5^ Pa·s), water (*ρ* = 997.05 kg m^−3^, *η* = 8.90 ×10^−4^ Pa·s), 4 M NaH_2_PO_4_ (*ρ* = 1313.00 kg m^−3^, *η* = 7.40 ×10^−3^ Pa·s), and 75% glycerol (*ρ* = 1191.95 kg m^−3^, *η* = 3.20 ×10^−2^ Pa·s) [28, 33, 34]. Our implementation of the Sader-Bellon model in Python is freely available, (see Data availability statement below).

### 2.2. Cantilever parameters

We modeled two silicon-nitride cantilevers relevant to SMFS experiments: a shorter, stiffer, and higher-frequency cantilever (BL-AC40TS; *k* = 90.0 pN nm^−1^, *L* = 38 *µ*m, *b* = 16 *µ*m, *h* = 0.2 *µ*m, *ρ* = 3170 kg m^−3^) and a longer, softer, and lower-frequency cantilever (MLCT-Bio B; *k* = 20.0 pN nm^−1^, *L* = 210 *µ*m, *b* = 20 *µ*m, *h* = 0.55 *µ*m, *ρ* = 3170 kg m^−3^). These geometries and material properties were used to compute the cantilever mass *m_c_* and, together with each medium’s density, the entrained fluid mass *m*_f_. These cantilever types were also used in the experimental calibration measurements described below, admitting comparison.

### 2.3. Resonance frequency estimation

While *k* is directly specified in equation (3) of the Sader-Bellon model, further analysis is needed to estimate the other SHO parameters of *Q* and *f*_0_. In this work we are particularly interested in estimating *f*_0_ to interpret and validate the iterative fitting strategy. In equation (3), the quantity ${k_n}-[{m_\mathrm{c}}+{m_\mathrm{f}}{\Gamma} (\omega )]{\omega^2}$ is the dynamic stiffness of mode *n* (recall that ${k_n} = \frac k{3}{C_n^4}$) [25]. Its real part vanishes when the elastic restoring force is balanced by the inertia of the cantilever and its entrained fluid, which approximates the condition of resonance and can be used to estimate *f*_0_ by numerically solving $\mathfrak{R}\{{k_n}-[{m_\mathrm{c}}+{m_\mathrm{f}}{\Gamma} (\omega )]{\omega^2}\} = 0$ for *ω*, using a bracketed root-finding algorithm (scipy.optimize.brentq). The initial bracket was centered on an estimate of the resonance frequency obtained by correcting the vacuum resonance frequency, ${\omega_{\mathrm{vacuum}}} = \sqrt{{k_n}/{m_\mathrm{c}}}$, for the real part of the added fluid mass evaluated at *ω*_vacuum_. The resulting estimate is:

\begin{equation*}{\omega_{0,\mathrm{guess}}} = \frac{{\omega_{\mathrm{vacuum}}}}{\sqrt{1+\frac{{m_\mathrm{f}}}{{m_\mathrm{c}}}\mathfrak{R}\left\{{\Gamma} \left({\omega_{\mathrm{vacuum}}}\right)\right\}}}.\end{equation*}

### 2.4. SHO fitting procedure

Several related approaches were used to fit the calculated PSDs with the SHO Lorentzian model of equation (2). Minimization was performed with differential evolution (scipy.optimize.differential_evolution), rather than a gradient-based method, to avoid sensitivity to the initial parameter guesses. In all fits, the model PSD was sampled on a uniform frequency grid with 38.15 Hz spacing, matching the frequency resolution of the experimental spectra. Fits were performed under four conditions, differing in whether the spectrum carried a detection-noise floor and in how the residuals were weighted. Residuals were computed either on linear PSD values, so the sum of the squared residuals is dominated by the high-amplitude points near the resonance peak, or on the logarithm of the PSD, which weights all sampled frequencies comparably. For the noiseless spectra, *S*_w_ was fixed at zero and the three remaining parameters (*f*_0_, *Q, k*) were fitted under both linear and logarithmic weighting. Spectra carrying noise were generated by adding a frequency-independent background of *S*_w_ = 2 ×10^−26^ m^2^ Hz^−1^ to the Sader-Bellon PSD, chosen to approximate the detection-noise level of the experimental thermal spectra, and were fitted with *S*_w_ as a fourth free parameter, under both weightings. Fits to experimental thermal spectra, which inherently contain a detection-noise floor, were always fitted with *S*_w_ as a free parameter.

In addition to differences in weighting and the inclusion of *S*_w_, we used both local (i.e. confined to a frequency range in the vicinity of the resonant peak) and global fitting strategies. In the local strategy, the fit window spanned $\left[ \frac{{f_{\mathrm{peak}}}}{\alpha},{f_{\mathrm{peak}}}\times \alpha \right]$, where the window half-width *α* sets how much of the spectrum on either side of the peak is included and *f*_peak_ is the numerical maximum of the first mode of the spectrum; *α* was varied systematically to find the optimum value (*α* = 3.5 for the noiseless spectra and *α* = 2.5 once a detection-noise floor was included; see sections 3.5 and 3.7). Because the apparent PSD peak can differ substantially from the *f*_0_ value identified from the dynamic-stiffness analysis (above) under strong damping conditions, the local fit was performed iteratively. After each fit, the window was recentered on the newly fitted *f*_0_ rather than on *f*_peak_ and the fit was repeated. We found that five iterations were always sufficient for convergence. In the global fit, a single fit was performed over a large fixed range from 10 Hz to 20 times the second-mode resonance frequency.

### 2.5. Bias computation

The accuracy of each fit was quantified as the percent bias in the recovered *k* relative to the input value used to generate the PSD, with positive values indicating overestimation of *k*:

\begin{equation*}\delta k = ~\frac{{k_{\mathrm{fit}}}-{k_{\mathrm{input}}}}{{k_{\mathrm{input}}}}\times 100\%.\end{equation*}

### 2.6. Experimental calibration

Experimental thermal calibration was performed on BL-AC40TS and MLCT-Bio B cantilevers using an Asylum Research (Oxford Instruments) Cypher ES AFM at 25 °C. For each cantilever, invOLS and thermal *k* were first calibrated in air using the instrument’s air holder. The cantilever was then transferred to a perfusion holder and the invOLS redetermined in Tris-buffered saline (20 mM Tris, 150 mM NaCl) before calibration in that medium. The invOLS was determined from force curves acquired against a freshly cleaved mica surface, with the trigger set to 1 V of deflection in both air and buffer. The slope was taken from a linear fit to the contact region of the retract curve, where the tip is in hard contact with the surface (figure 1(b)). In each medium, five replicate calibrations were performed. Each replicate consisted of a new invOLS determination followed by acquisition of a thermal spectrum, and each spectrum was converted to displacement units using its own paired invOLS value (a one-to-one pairing). For each calibration, the thermal PSD was computed from consecutive 26.2 ms deflection time records, each Fourier-transformed to give a spectrum with 38.15 Hz frequency resolution. Approximately 250 such spectra were averaged to reduce the statistical scatter of the PSD. The spectra extended from 38.15 Hz to 8 MHz, except for the MLCT-Bio B in air, which extended to 2 MHz. Data were fit to the SHO model with *S*_w_ term (equation (2)) and logarithmic weighting using the local strategy with α = 2.5. Fitting was performed using the same custom Python routines applied to the theoretical data to allow direct comparison.

The replicate variance of *k* was separated into contributions from thermal PSD fitting and invOLS variability. The statistical uncertainty of each fitted *k* was estimated from the parameter covariance matrix of the final log-weighted fit. These variances were averaged and the remaining replicate variance, attributed to variability in determining invOLS, was obtained by subtracting the fitting variance from the total variance of *k*, so that the two contributions combine in quadrature to give the standard deviation reported in table 3.

## 3. Results and discussion

### 3.1. A physically realistic PSD, fit with the experimentally relevant SHO model

To probe how the choice of fitting strategy affects calibration accuracy, we generated thermal displacement PSDs using the Sader-Bellon model, which captures the frequency-dependent hydrodynamic loading experienced by a cantilever oscillating in a viscous fluid. Unlike the SHO approximation, the resulting PSD lacks a flat, white-noise plateau at low frequency and falls off more gradually than *f*^−4^ at high frequency. Despite this, we fit each model PSD with the same SHO Lorentzian model that experimentalists routinely use to calibrate real cantilevers, regardless of the medium (equation (2)). Sader showed that in the limit of small dissipation and in the vicinity of a resonance peak, the frequency response of a cantilever in fluid reduces analytically to that of a SHO, with *f*_0_ and *Q* given by equations 33 and 35 of Reference 22. Thus, at least for sufficiently weak damping and for a sufficiently narrow frequency range around *f*_0_, equation (2) is expected to adequately describe the shape of the hydrodynamically realistic PSD. One question remains how rapidly it ceases to be adequate as damping increases, as in liquid.

This strategy was applied to two cantilevers relevant to SMFS experiments: a stiff, shorter cantilever (BL-AC40TS) [35] and a soft, longer cantilever (MLCT-Bio B), [36] each modeled in four media: air, water, 4 M NaH_2_PO_4_, and 75% glycerol. The latter two media were chosen to admit comparison with the study of Pirzer and Hugel [28]. For each cantilever-medium combination, equation (2) was fit using two strategies: a local fit restricted to a window around the resonance peak and a global fit spanning the full measured spectrum. The local fit was performed iteratively, recentering the fit window on the newly recovered resonance frequency until convergence. The consequences of fitting only once, without this recentering, were also examined separately.

### 3.2. Local fitting near the resonance peak accurately recovers the spring constant across all media

Fitting the SHO Lorentzian [equation (2), with *S*_w_ = 0] to PSDs predicted by the Sader-Bellon model in a local window centered on the resonance peak ($\left[ \frac{{f_{\mathrm{peak}}}}{\alpha},{f_{\mathrm{peak}}}\times \alpha \right]$, with *α* = 3.5) recovered the input *k* with low bias across all four media tested, for both cantilevers. For the stiff cantilever (BL-AC40TS), the local-fit *k* bias was +2.4% in air, +1.9% in water, +1.9% in 4 M NaH_2_PO_4_, and −0.5% in 75% glycerol (table 1; figure 3). For the soft cantilever (MLCT-Bio B), the corresponding biases were +2.3%, +2.4%, +0.3%, and −2.1% (table 1; figure S2). In both cases, the bias remained below roughly 2.4% in magnitude even as the quality factor of the fundamental mode fell from *Q* ≈ 18–27 in air to as low as *Q* ≈ 0.4 in glycerol (i.e. overdamped). The Sader-Bellon PSD departs from the SHO shape in every medium, lacking the flat low-frequency plateau and the *f*^−4^ rolloff that the SHO assumes. What changes with increasing viscosity is how much of the spectrum lies in these mismatched regions relative to the resonance itself (figure 3).

**Figure 3.**
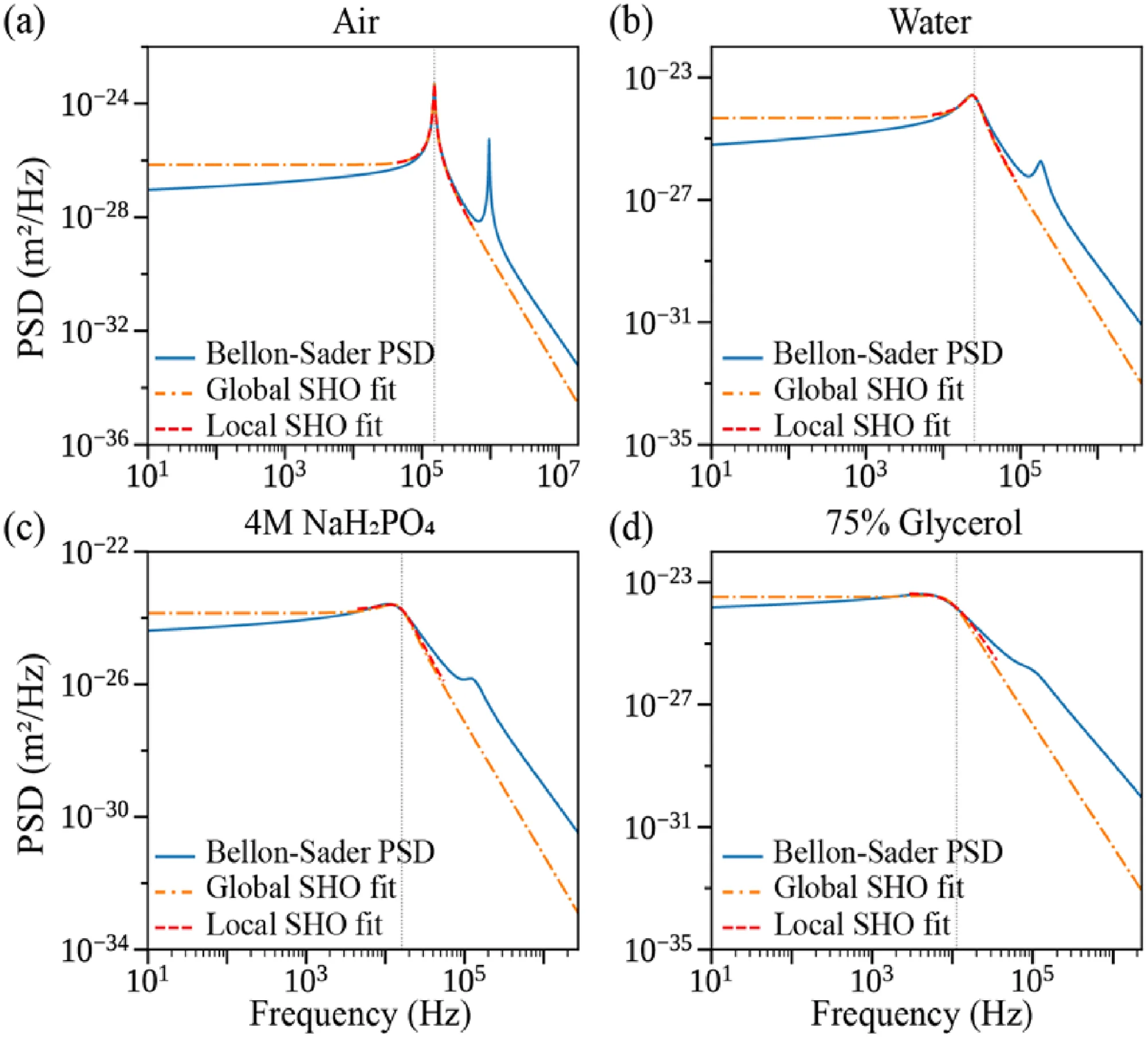
Modeled thermal noise PSD of the BL-AC40TS cantilever (blue) in (a) air, (b) water, (c) 4 M NaH_2_PO_4_, and (d) 75% glycerol, with the best-fit single-mode SHO Lorentzian (equation (2) with *S*_w_ = 0) obtained using the local (red) and the global (orange) fitting strategies. The local fit was performed iteratively, restricting the fit to a window of half-width *α* = 3.5 around the resonance peak spanning $\left[ \frac{{f_{\mathrm{peak}}}}{3.5},{f_{\mathrm{peak}}}\times 3.5\right]$ and recentering on the recovered *f*_0_ over five iterations; the global fit was a single fit spanning the full spectrum. The gray dotted vertical line marks the fluid-loaded resonance frequency *f*_0_ of the fundamental mode. Note that each fit is drawn only over the frequency range on which it was performed. The local fit would also depart from the model PSD if extrapolated to a wider frequency range.

**Table 1.** Local and global SHO fit parameters for the modeled BL-AC40TS and MLCT-Bio B cantilevers across four media of increasing viscosity, obtained with residuals computed on linear PSD values. The local fit was performed iteratively with *α* = 3.5 while the global fit spanned the full spectrum.

Medium	Local fit				Global fit			
	*f*_0_ (kHz)	*Q*	*k* (pN nm^−1^)	δ*k* (%)	*f*_0_ (kHz)	*Q*	*k* (pN nm^−1^)	δ*k* (%)
*BL-AC40TS (k = 90.0 pN nm^−1^)*								

Air	151.9	26.5	92.1	+2.4	151.9	26.5	92.1	+2.4
Water	25.2	2.2	91.7	+1.9	25.1	2.3	95.2	+5.8
4 M NaH_2_PO_4_	15.5	1.1	91.7	+1.9	15.0	1.2	100.4	+11.5
75% glycerol	10.2	0.6	89.5	−0.5	9.1	0.8	102.2	+13.5

*MLCT-Bio B (k = 20.0 pN nm^−1^)*								

Air	16.5	18.1	20.5	+2.3	16.5	18.1	20.5	+2.3
Water	3.2	1.3	20.5	+2.4	3.2	1.5	22.0	+9.9
4 M NaH_2_PO_4_	1.6	0.7	20.1	+0.3	1.5	0.9	22.7	+13.7
75% glycerol	0.9	0.4	19.6	−2.1	0.7	0.7	22.8	+14.1

δ*k* denotes the percentage bias of the fitted *k* relative to the input *k* (equation (6)).

These fits used residuals computed on linear PSD values. Repeating them with residuals computed on the logarithm of the PSD degrades the recovered *k* substantially, and progressively so with damping. The local-fit bias goes from +6.6% in air to −25.9% in 75% glycerol for BL-AC40TS, and from +4.9% to −36.8% for MLCT-Bio B (table S1). Logarithmic weighting distributes the fit across all sampled frequencies, drawing it toward the off-resonance regions where the Sader-Bellon and SHO lineshapes diverge, which is precisely what the local window is intended to exclude.

### 3.3. Global fitting degrades as viscosity increases

In contrast to the local fit, fitting the SHO Lorentzian with linear weighting over a global range spanning the full measured spectrum produced low bias only in air, with progressively larger bias once the cantilever was immersed in liquid. For the BL-AC40TS cantilever, the linear global fit *k* bias was +2.4% in air, +5.8% in water, +11.5% in 4 M NaH_2_PO_4_, and +13.5% in 75% glycerol, while for the MLCT-Bio B cantilever, the bias was +2.3% in air, and +9.9%, +13.7%, and +14.1% across the same media (table 1). The air panel of figure 3 shows the global fit can be accurate in *k* while being visibly misaligned with much of the spectrum. The fitted curve overestimates the low-frequency plateau by two orders of magnitude, but those points contribute negligibly to a linear residual sum, so the recovered *k* is set almost entirely by the peak. As viscosity increases, the mismatched regions move closer to the peak in amplitude and can no longer be ignored.

Under logarithmic weighting, the global fit fails outright, returning *Q* below 1 in every medium and *k* overestimated by up to a factor of ∼13 (table S1). With all frequencies weighted comparably, the fit is forced to accommodate the low-frequency plateau and the shallow high-frequency rolloff that the SHO form cannot reproduce.

### 3.4. Iterative fitting is needed in more-viscous media

The local fits presented in figure 3 were performed iteratively. The fit window was recentered on the newly recovered *f*_0_ after each fit, for a total of five iterations, to correct for cases in which the apparent peak of the PSD differs from the fluid-loaded *f*_0_. This recentering proves unnecessary in some conditions but essential in others. When the local fit is performed only once, with the window centered on the raw PSD peak, the recovered *k* for the stiff cantilever (BL-AC40TS) remains accurate in air (+2.4%) and water (+2.2%), with the departure from the iterative result growing in 4 M NaH_2_PO_4_ (+4.7%) and becoming large in 75% glycerol (+10.5%) (figure 4). The soft cantilever (MLCT-Bio B) exhibited the same breakdown but more severely, with non-iterative bias reaching +10.0% in 4 M NaH_2_PO_4_ and +21.3% in 75% glycerol (figure S3). This is consistent with its lower *Q* and corresponding larger separation between the apparent peak and the actual resonance, leading the window to be centered on a frequency well below *f*_0_. Iterative recentering was therefore adopted as the standard local fitting procedure throughout this work.

**Figure 4.**
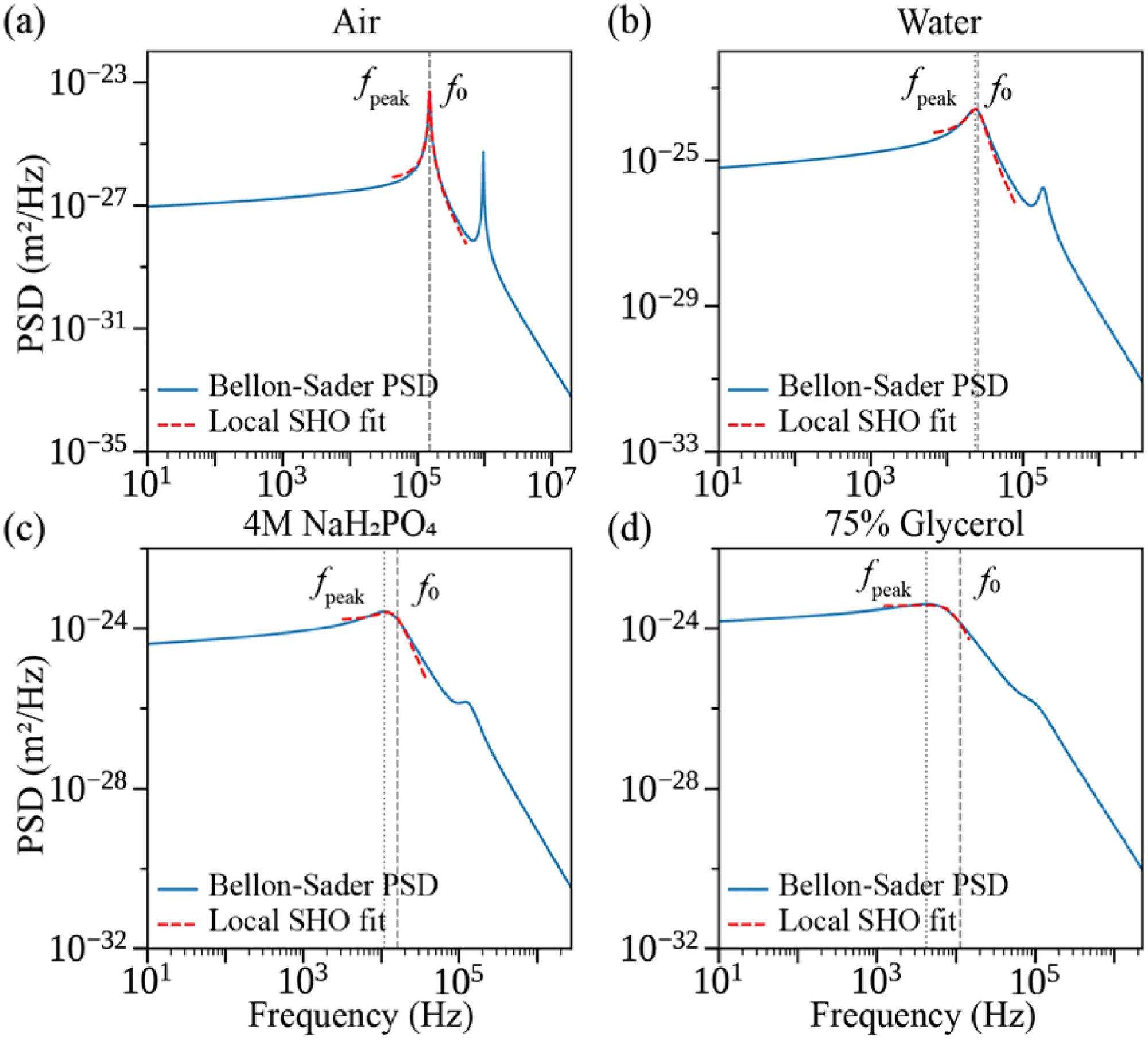
Modeled thermal noise PSD of the BL-AC40TS cantilever (blue) with a non-iterative local SHO fit (red; window half-width *α* = 3.5), shown for (a) air, (b) water, (c) 4 M NaH_2_PO_4_, and (d) 75% glycerol. The dotted gray line marks *f*_peak_, the apparent maximum of the PSD used to center the fit window, and the dashed gray line marks *f*_0_ calculated as discussed in section 2.3.

These results bear directly on the conclusions of Pirzer and Hugel, who found experimentally that thermal calibration in 75% glycerol deviated by up to 50% from that in water and concluded that the method was unreliable in media of high viscosity [28]. Their fits extended from the lowest sampled frequency to the minimum between the first two flexural modes, and the recovered *k* was obtained by integrating the fitted function to give $\langle {x^2}\rangle$. Both features make the result sensitive to the off-resonance regions where the SHO lineshape departs most strongly from the true response. In fact, their analysis showed the recovered *k* varied by more than 50% as the lower limit of the fit range was moved. Our global-fitting results similarly show that large biases can be obtained under certain fitting conditions (table 1), but also that restricting the fit to a window around the resonance and recentering that window iteratively removes most of that bias. We note that the comparison with Pirzer and Hugel is not exact, since their values were obtained from experimental spectra and include additional contributions from optical lever sensitivity and noise.

### 3.5. An intermediate fit window width minimizes local fit bias

The iterative local fitting results used a window half-width *α* = 3.5, spanning $\left[ \frac{{f_{\mathrm{peak}}}}{3.5},{f_{\mathrm{peak}}}\times 3.5\right]$ around the recentered peak. To assess the sensitivity of the results to this choice, we carried out the iterative local fit for the stiff cantilever (BL-AC40TS) across values of *α* = 1.5–5.0 in steps of 0.5 in all four media. In air, absolute δ*k* was essentially insensitive to *α*, remaining near 2.3% across the entire range. In the three liquid media, however, the absolute bias traced out a clear U-shaped dependence on *α*. Bias was large at small *α*—up to 17% at *α* = 1.5 in glycerol—passed through a minimum whose location shifted to larger *α* with increasing viscosity (near *α* = 2.5 in water, 3.0 in 4 M NaH_2_PO_4_, and 3.5 in 75% glycerol), and rose again at larger *α*, reaching 3%–5% by *α* = 5.0 (figure 5). At *α* = 3.5, the bias remained below 3% in all media.

**Figure 5.**
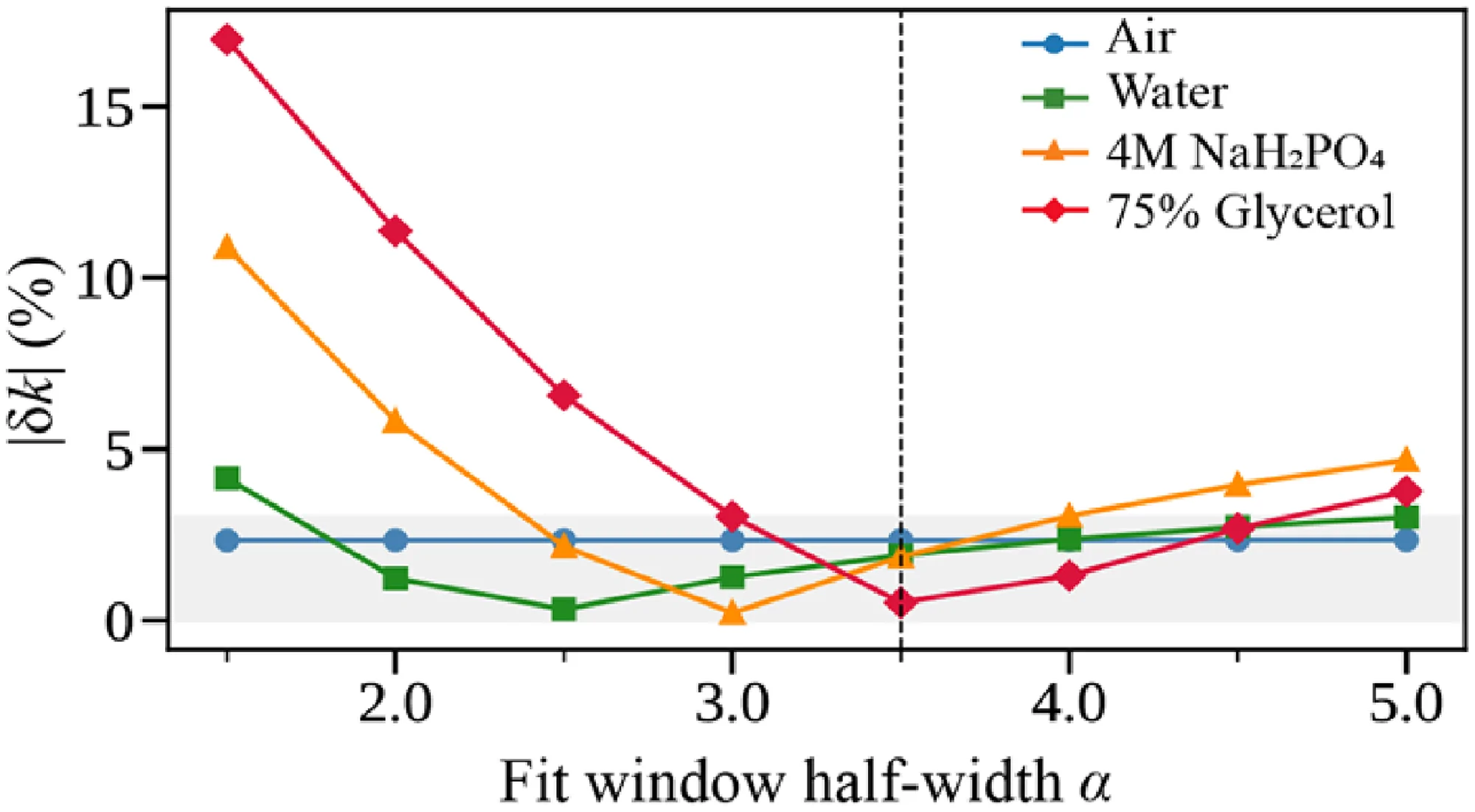
Absolute bias in the iterative local-fit spring constant, |δ*k*|, as a function of the fit window half-width *α*, for the BL-AC40TS cantilever in air, water, 4 M NaH_2_PO_4_, and 75% glycerol, using the noiseless Sader–Bellon PSD. The fit window spans [*f*_peak_/*α*, *f*_peak_ × *α*] around the iteratively recentered peak. The bias is minimized near *α* = 3.5 (dashed vertical line), where it remains below 3% (gray shaded band) in all four media.

This non-monotonic dependence reflects two competing failure modes of the local fit. At small *α*, the window is too narrow to resolve the width and shape of the resonance peak, particularly once that peak is broad and asymmetric under strong damping. The fit becomes underdetermined and increasingly biased. At large *α*, the window extends into the off-resonance wings, where the Sader-Bellon PSD departs most strongly from the SHO expectation. An intermediate *α* balances these two effects across all media.

The same sweep performed for the MLCT-Bio B cantilever showed the same U-shaped dependence, but with minima displaced to somewhat larger *α* (figure S4). At *α* = 3.5, the bias remains within 3% in all media for the soft cantilever as well, so the same window width serves both cantilevers. This illustrates a more general point that the optimal window width is not universal but depends on the cantilever and the medium, through the sharpness of the resonance. For experimental work in a particular cantilever-medium combination, the window width can be optimized directly by repeating the sweep presented here with the relevant cantilever dimensions and fluid properties using our freely available analysis code accompanying this work (see Data availability statement below).

### 3.6. Fitting spectra with a noise floor requires logarithmic weighting

The analysis above used noiseless spectra, representing only the thermal motion of the cantilever; experimental spectra sit on a detection-noise floor that can be modeled using the *S*_w_ term in equation (2) and that could alter the fitting strategy. To determine how the floor affects the choice of weighting, Sader-Bellon PSDs were regenerated with a white-noise floor of *S*_w_ = 2 ×10^−26^ m^2^ Hz^−1^ and fitted with the extended SHO model of equation (2) (i.e. including *S*_w_ as a fit parameter), using both linear and logarithmic residuals under otherwise identical conditions.

With the noise floor present, the two weightings diverge sharply for the local fit (table 2). Under linear weighting, the recovered *k* is biased by up to 13% in the viscous media for both cantilevers and the fitted noise floor is overestimated by roughly an order of magnitude for the BL-AC40TS cantilever and by 2–3 orders of magnitude for the MLCT-Bio B cantilever. For the MLCT-Bio B in air, the linear fit does not constrain *S*_w_ at all. Logarithmic weighting recovers *k* to within about 3% in every medium and, while it does not always accurately recover *S*_w_, it constrains it well enough that *k* remains accurate (table 2; fits shown in figures S5 and S6). Full results for all four fit parameters are given in table S3. These biases were insensitive to the cantilever parameters assumed in the model. Concerted variation of the cantilever mass, thickness, width, and hydrodynamic loading in ways expected not to change *k* affected its recovered value by at most 0.3 percentage points (supplementary note 1). The biases are also insensitive to contamination from higher-order flexural modes, which lie well above the local fit window in any medium. Removing the second mode, which was included in all PSDs in this work, changed the recovered *k* by no more than 0.2 percentage points. Global fitting of the entire PSD gives poor results with both weightings (table S2). Thus, when fitting noise-containing data—including experimental data—we used the iterative local fit with logarithmic weighting.

**Table 2.** Local fits of the SHO model with *S*_w_ as a fit parameter using linear and logarithmic residual weighting, applied to Sader-Bellon PSDs with 2 × 10^−26^ m^2^ Hz^−1^ added white noise. All fits were iterative with *α* = 2.5.

Medium	Linear weighting		Logarithmic weighting	
	δ*k* (%)	*S*_w_ (m^2^ Hz^−1^)	δ*k* (%)	*S*_w_ (m^2^ Hz^−1^)
*BL-AC40TS (k = 90.0 pN nm^−1^)*				

Air	+2.3	2.0 × 10^−26^	+2.9	2.0 × 10^−26^
Water	+5.2	7.2 × 10^−26^	+0.6	4.2 × 10^−26^
4 M NaH_2_PO_4_	+10.0	1.5 × 10^−25^	−1.4	7.3 × 10^−26^
75% glycerol	+10.9	2.2 × 10^−25^	−2.1	1.1 × 10^−25^

*MLCT-Bio B (k = 20.0 pN nm^−1^)*				

Air	+2.2	n.c.^a^	+3.1	3.0 × 10^−26^
Water	+9.0	2.7 × 10^−24^	−1.1	1.0 × 10^−24^
4 M NaH_2_PO_4_	+11.6	5.8 × 10^−24^	−2.0	2.5 × 10^−24^
75% glycerol	+13.1	1.1 × 10^−23^	+0.1	5.0 × 10^−24^

^a^ n.c. = not constrained.

We argue that logarithmic weighting is more effective for the noise-containing data because the white-noise floor lies far below the resonance peak in the off-resonance region of the spectrum (figures S5 and S6). A fit with linear weighting is dominated by the high-amplitude points near the peak, leaving *S*_w_ effectively unconstrained. Failure to constrain one fit parameter can bias the other parameters, including *k.* Logarithmic weighting gives the off-resonance points comparable influence, better constraining the noise floor. This is the opposite of the noiseless case, where the off-resonance region is where the functional forms of the SHO and Sader-Bellon models diverge.

### 3.7. Detection noise reshapes the window tradeoff

The presence of a white-noise floor could in principle shift the optimal *α* for local fitting. To test this, we repeated the fit-window sweep of figure 5, now fitting to Sader-Bellon PSDs with 2 ×10^−26^ m^2^ Hz^−1^ added frequency-independent noise using equation (2) with logarithmic weighting and *S*_w_ as a fourth fit parameter.

For the BL-AC40TS cantilever, the noise floor reshaped the individual |δ*k*| vs α curves; now *α* = 2.5 provided sub-3% bias across media (figure 6). As in figure 5, the bias for all media except air was large at small α because of poor sampling of the resonance peak. As *α* was increased, the bias initially decreased, passing through a minimum, then increased again in the more viscous media because the flat noise floor—rather than the mismatched Sader-Bellon wings—now dominated the off-resonance region. The same sweep for the MLCT-Bio B cantilever shows that *α* = 2.5 is likewise a good value (figure S7).

**Figure 6.**
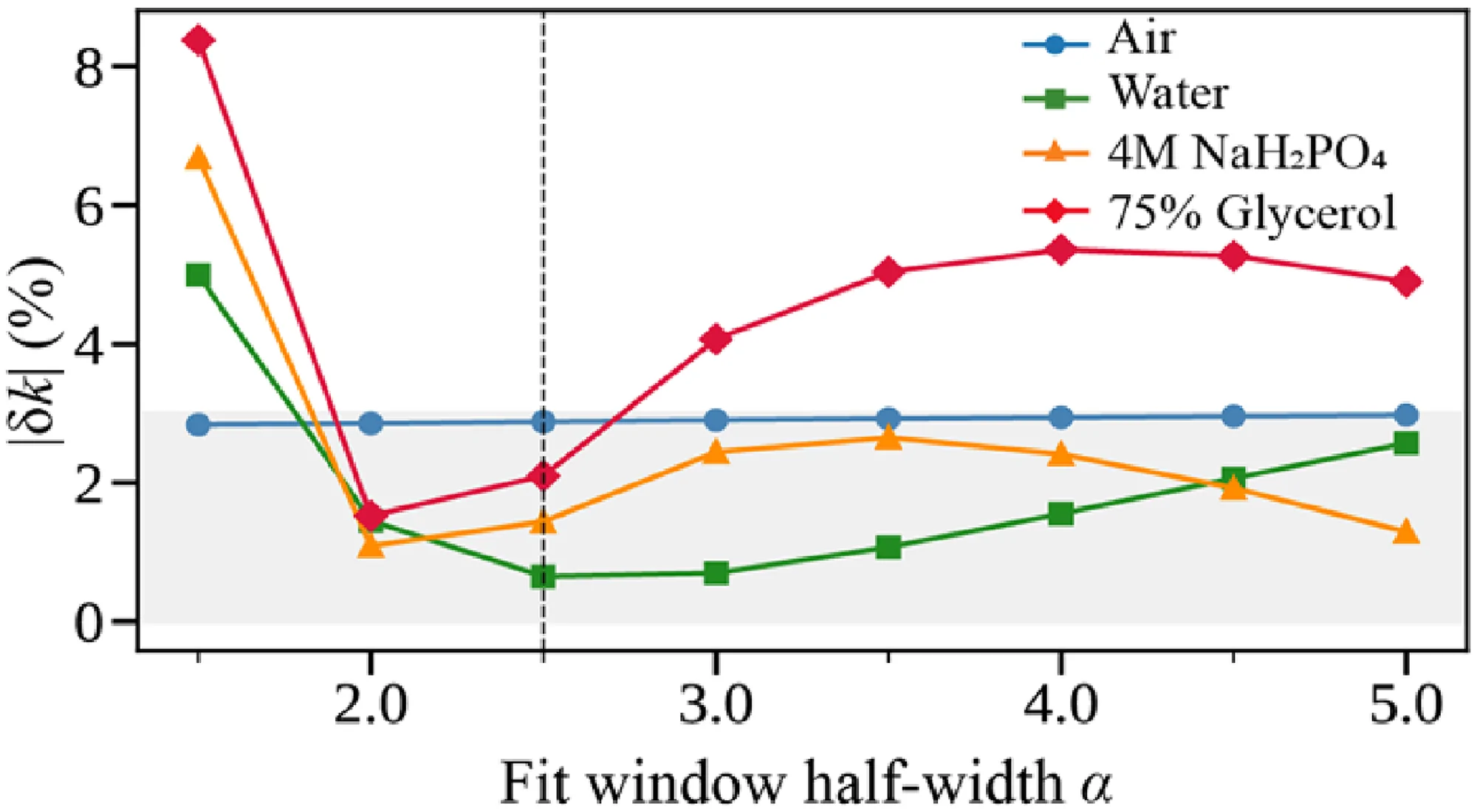
Robustness of the fit-window choice to detection noise. The *α* sweep in figure 5 was repeated for the BL-AC40TS cantilever after adding a white-noise floor (*S*_w_ = 2 × 10^−26^ m^2^ Hz^−1^) to the Sader-Bellon PSD, representative of the detection-noise level of a typical AFM. Each spectrum was now fit with the noise-containing SHO model (equation (2)), with *f*_0_, *Q, k*, and *S*_w_ as fit parameters and logarithmic weighting.

### 3.8. *In situ* calibration in buffer agrees with calibration in air

Having used theoretical spectra to optimize a calibration strategy based on iterative local (α = 2.5) fitting of the four-parameter SHO model (equation (2), including noise term) with logarithmic weighting, we sought to test this approach by fitting experimental thermal spectra of the two cantilevers measured in air and buffer (20 mM Tris HCl, 150 mM NaCl). Immersion in buffer strongly alters the thermal PSD of both cantilevers (figure 7). For the BL-AC40TS, *f*_0_ shifts from 93.8 kHz in air to 28.0 kHz in buffer and *Q* falls from 37.6 to 1.6 (table 3). The softer MLCT-Bio B is affected more severely, its *f*_0_ dropping from 13.8 kHz to 3.2 kHz and *Q* from 8.1 to 0.6, close to the critical damping condition (table 3). These shifts are the expected consequences of fluid loading, with entrained mass lowering *f*_0_ and viscous drag reducing *Q*.

**Figure 7.**
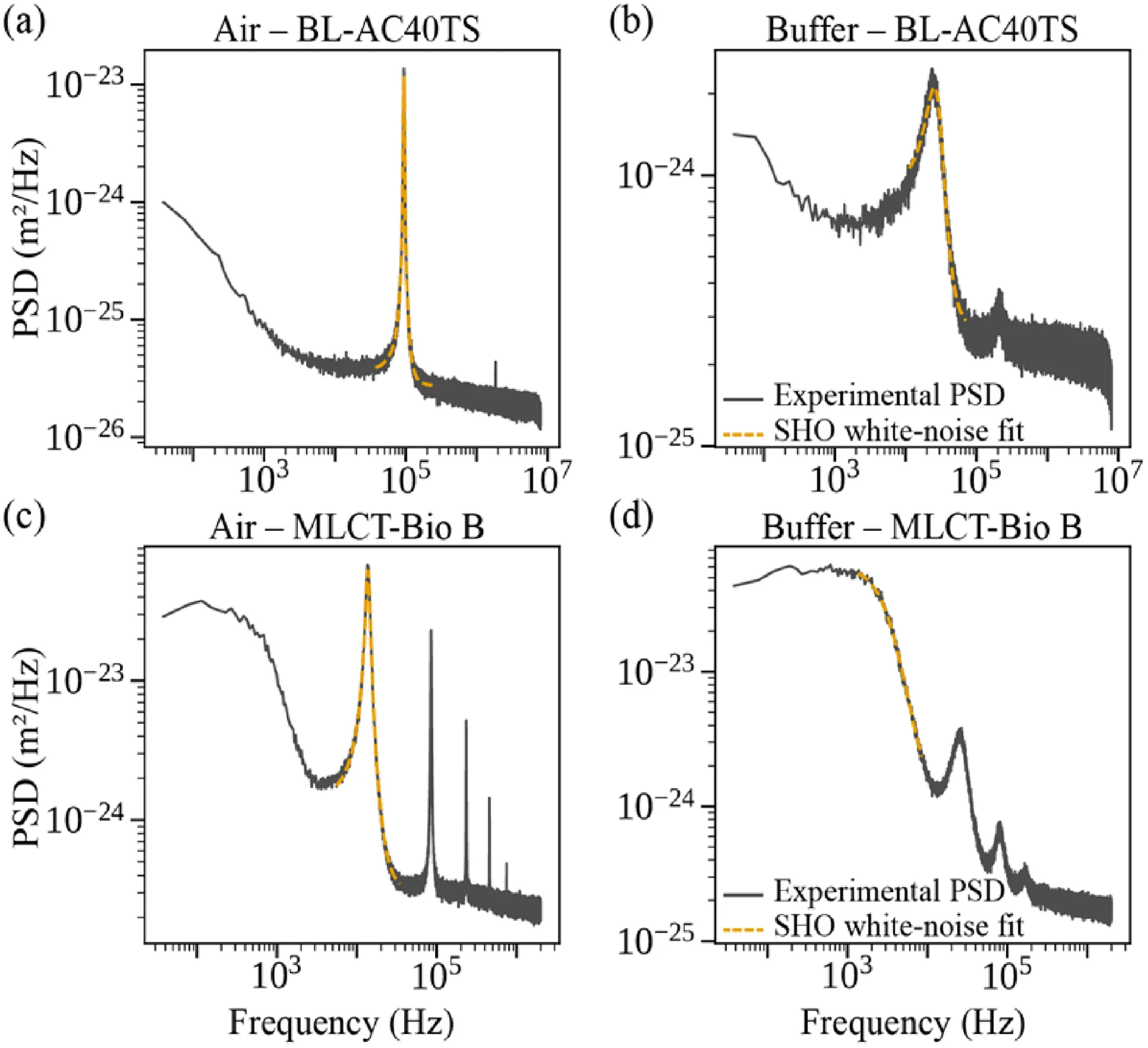
Experimental application of the optimized fitting strategy to two types of cantilevers. Experimental thermal noise PSDs (black) of freely oscillating cantilevers above a mica substrate were fit (orange) with the four-parameter SHO model (equation (2), including *S*_w_ term) using *α* = 2.5 and logarithmic weighting. Shown are representative results for the stiffer BL-AC40TS cantilever in (a) air and (b) buffer and for the softer MLCT-Bio B cantilever in (c) air and (d) buffer.

**Table 3.** Experimental thermal calibration of the BL-AC40TS and MLCT-Bio B cantilevers in air and buffer. Spectra were analyzed with the four-parameter SHO model (equation (2), including *S*_w_ term) using α = 2.5 and logarithmic weighting. Each entry is the mean ± standard deviation of five replicate measurements of the same cantilever; each thermal spectrum was paired with its own independent invOLS determination.

Cantilever	Medium	*f*_0_ (kHz)	*Q*	*k* (pN nm^−1^)^a^	*S*_w_ (m^2^ Hz^−1^)
BL-AC40TS	Air	93.8 ± 0.01	37.6 ± 1.6	93.7 ± 3.5	(2.65 ±0.10) × 10^−26^
	Buffer	28.0 ± 0.07	1.6 ± 0.1	93.4 ± 1.0	(2.66 ±0.03) × 10^−25^
MLCT-Bio B	Air	13.8 ± 0.01	8.1 ± 0.6	24.3 ± 0.4	(3.08 ±0.05) × 10^−25^
	Buffer	3.2 ± 0.06	0.6 ± 0.1	23.7 ± 0.9	(6.15 ±0.28) × 10^−24^

^a^ The estimated uncertainties from thermal PSD fitting and invOLS variability, respectively, were: BL-AC40TS air, 0.4 and 3.5 pN nm^−1^; BL-AC40TS buffer, 0.3 and 1.0 pN nm^−1^; MLCT-Bio B air, 0.1 and 0.4 pN nm^−1^; MLCT-Bio B buffer, 0.7 and 0.6 pN nm^−1^.

Despite pronounced changes in *f*_0_ and *Q*, the recovered *k* values were nearly unchanged between the two media, consistent with the understanding of *k* as a medium-independent material property of the cantilever. As seen in table 3, we measured *k* values for the stiffer BL-AC40TS cantilever in buffer that agreed within 1% of its value in air and for the softer MLCT-Bio B that agreed within 3%. Both are consistent with the few-percent differential bias predicted by our modeled analysis (table 2). The small residual differences are compatible with the additional experimental uncertainty in invOLS calibration, which must be redetermined in each medium and can be particularly challenging in air because of unscreened electrostatic interactions between the probe and the substrate, as illustrated in figure S1.

## 4. Conclusion

We have used a physically realistic hydrodynamic model of cantilever thermal motion to test how the choice of fitting strategy affects thermal calibration of the spring constant across fluid viscosities spanning more than three orders of magnitude for two cantilever types directly relevant to SMFS experiments. We determined that, in the presence of white noise typical of experimental data, the best calibrations could be obtained by fitting a four-parameter SHO model (equation (2), including noise term *S*_w_) in an iterative manner in the local vicinity of the first resonant peak (half-width *α* = 2.5, the optimum in the presence of noise) using logarithmic weighting. Application of this model to theoretical PSDs yielded *k* biases of <3% in water for both cantilever types; application of the model to experimental data showed agreement between air and buffer calibrations at the level of 1% for the stiffer cantilever and 3% for the softer cantilever. Taken together, these results indicate that AFM cantilevers intended for liquid-phase single-molecule measurements can be calibrated reliably *in situ* without the need for a separate calibration in air if appropriate fitting conditions are used. For cantilevers with substantially different properties from those investigated here, this conclusion can be checked and the fitting strategy perhaps refined by repeating our analysis with new parameter values using the same simulation code we have made freely available.

## Data Availability

The data and software that support the findings of this study are openly available at the following URL/DOI: https://github.com/JacobsonLabClemson/afm-cantilever-calibration-viscosities. Supplemental Information available at https://doi.org/10.1088/1361-6501/aebb53/data1.
